# Recovery of Male Siamese Fighting Fish (*Betta splendens*) After Overland Shipping

**DOI:** 10.3390/ani15142156

**Published:** 2025-07-21

**Authors:** Karun Thongprajukaew, Saowalak Malawa, Sukanya Poolthajit, Nutt Nuntapong, Waraporn Hahor

**Affiliations:** 1 Division of Health and Applied Sciences, Faculty of Science, Prince of Songkla University, Songkhla 90110, Thailand; poolthajit123@gmail.com (S.P.); waraporn.hahor@gmail.com (W.H.); 2 Faculty of Agricultural Technology, Songkhla Rajabhat University, Songkhla 90000, Thailand; saowalak.ma@skru.ac.th; 3 Aquatic Science and Innovative Management Division, Faculty of Natural Resources, Prince of Songkla University, Songkhla 90110, Thailand; nutt.n@psu.ac.th

**Keywords:** fish packaging, live fish transportation, recovery time, weight loss, welfare

## Abstract

Ornamental fish shipped by road or rail may spend days in transit without food, leading to a reduction in somatic growth after transportation and during acclimatization. In the present study, a time-series (0, 2, 4, 6, 8, 10, and 12 days) experiment was conducted to investigate the growth recovery of male Siamese fighting fish (*Betta splendens*, 1.56 ± 0.02 g body weight, *n* = 15 per group) transported by road for two days. Biometric changes, nesting activity, skin pigmentation, digestive enzyme activity, muscle quality, and whole-body composition, were compared across all fish groups. The recovery in growth, as indicated by final body weight, increased with post-transportation time (*p* < 0.05), causing a significant reversal of weight loss with a proportionally stable condition factor from day 8 until the end of observation (*p* > 0.05). During this time period, the fish exhibited similar bubble-nest building activity to the control group that was not transported (*p* > 0.05). Color parameters, digestive enzyme activities, muscle quality, and whole-body composition of fish 8 days after shipping were comparable to the control fish group (*p* > 0.05). Our findings indicate that an 8-day recovery time is an appropriate protocol for Siamese fighting fish acclimatization following overland shipping.

## Simple Summary

Siamese fighting fish are economically valuable ornamental fish. Commercial overland transportation of this fish species is widespread, although little is known regarding the growth recovery period following the journey. The present research investigated a 12-day recovery experiment following two days of overland transit. Based on biometric changes, nesting activity, skin pigmentation, digestive enzyme activity, muscle quality, and whole-body composition, eight days is a reasonable period of time to restore somatic growth. Our results offer clues for the proper post-transport treatment of this species. Transportation and post-transport acclimatization protocols might be predicated on an 8-day recovery period.

## 1. Introduction

The Siamese fighting fish (*Betta splendens*), Thailand’s national aquatic animal, is admired globally for its aggressive behavior, variety of colors, and physical traits. These qualities have led to steady growth in the popularity of the species, which is sold through various channels to hobbyists and pet stores inside and outside Thailand, and consistently ranks among the top two ornamental fish for market share in Thailand [1,2].

Shipping may have an effect on the health and welfare of live ornamental fish [3]. Since online sales are rapidly growing in Thailand, fish are usually transported overland via the country’s well-established postal system [4]. Commercial flights are usually deployed to countries outside of Thailand [3]. In general, male fish are transported individually to avoid the stressful aggression they show toward other males that may lead to injuries [4,5]. They are individually shipped in bags that contain approximately 150 mL of water [6] giving a water–air ratio of 1:3 or 3:5 by volume [7]. Thongprajukaew et al. [4] reported that 80 mL of water was adequate for domestic transportation of mature male Siamese fighting fish (1.44–1.50 g).

Normally, ornamental fish receive no food during transportation, which negatively affects their metabolism and health status [8,9,10]. Although limiting feed or fasting can negatively affect growth and health, the transported fish can recover their biometrics when normal feeding is resumed [11,12]. Changes in feed intake and nutritional response can be tracked by digestive enzymes that break down proteins (pepsin, trypsin, and chymotrypsin), lipids (lipases), and carbohydrates (amylase) to provide energy for homeostasis or recovery growth [4]. However, growth recovery after periods of starvation or food scarcity may be different depending on the species, age, and size of fish.

In ornamental fish, prolonged stress from the movement of the vehicle and the vibration of the water during transportation altered the skin coloration [4]. The stress responses from these stimuli may also be linked to cortisol, which affects the ability of male fighting fish to form bubble-nests [13]. Therefore, the recovery of fighting fish can be investigated using such parameters. In addition, since white muscle contributes significantly to a fish body mass and is essential to growth, the mechanisms governing growth can be more clearly recognized by investigating the rates of protein synthesis and degradation in white muscle [14,15,16]. The two major myofibrillar proteins, actin and myosin, are crucial for the contraction of muscles during transportation and rearing, allowing fish to swim, maintain posture, and perform other movements [17,18]. Both muscle measurements are therefore sensitive enough to measure physiological responses while recovering.

There are very few studies on growth recovery in aquatic animals after transportation. After being transported for 3 h, juvenile European eels (*Anguilla anguilla*) can recover plasma cortisol in 72 h [19], while Nile tilapia fingerlings (*Oreochromis niloticus*) can accomplish this within 48 h [20]. Cortisol and glucose have to be recovered within 72 h for juvenile Pacific bluefin tuna (*Thunnus orientalis*) after 45 min of driving and 10 min of boating [21]. There have been no reports of longer-term research examining the body’s growth response. Since the recovery of Siamese fighting fish after transportation has never been assessed, a practical protocol for commercial enterprises and hobbyists does not exist. Fish that have been deprived during transportation might benefit from being fed again to help them physically recover. Therefore, the objective of the current study was to investigate the recovery time of male Siamese fighting fish after overland transportation by their biometric changes, nesting activity, skin coloration, digestive enzyme activity, muscle quality, and whole-body composition.

## 2. Materials and Methods

### 2.1. Ethics of Animals

The transportation, acclimatization, rearing, sampling, and euthanasia of the fish conformed to the “Ethical Principles and Guidelines for the Use of Animals for Scientific Purposes” of the National Research Council of Thailand (Application No. U1-06514-2560).

This study was approved by the Prince of Songkla University Institutional Animal Care and Use Committee (Project Code 2564-01-101, approval date: 1 April 2022).

### 2.2. Fish Preparation

Solid-red, short-finned, 3.5-month-old male Siamese fighting fish (*n* = 150) were obtained from Kanasanan Betta Farm in Nakhon Pathom Province, Thailand. During their 10-day acclimatization, the fish were housed individually in 150 transparent polypropylene cups measuring 9.0 cm in diameter × 11.5 cm in height containing 350 mL of water (5.50 cm in depth). The fish were given a commercial diet made specifically for fighting fish (Betta-Bio Gold; Kyorin Food, Himeji, Japan) which included 40% crude protein twice a day (8.30 and 16.30 h). For ten days, the fish were housed in a 12 h light/12 h dark photoperiod. During this time, 80% of the water in the cups was replaced every other day with stock water that had been dechlorinated.

### 2.3. Fish Packaging and Transportation

One hundred and twenty fish were screened by size (1.56 ± 0.02 g body weight, 5.91 ± 0.04 cm total length) and starved for 12 h. As a control group, non-transported fish (*n* = 15) were raised in 150 mL clear polypropylene cups. The other fish (*n* = 105) were then transferred to white polyethylene bags measuring 10 cm in width × 31 cm in height, containing 80 mL of water with 6.5 mg L^−1^ of methylene blue [4]. Each bag contained a 1:3 water to air ratio. The packaging technique adhered to the Thai Post Office’s typical operating practice. Five polyethylene bags were wrapped in double-layer packaging and bubble wrap and packed in a paper box (20.5 cm width × 29.5 cm length × 11.0 cm height). Twenty-one boxes were packed with five bags giving a total of 105 shipped fish. All the boxes were shipped by road from Prince of Songkla University (PSU, starting time = 9.00 h), Hat Yai Campus, to a destination in Suratthani Province, Thailand, and then returned to PSU, Hat Yai Campus. During transportation, the humidity (57.6–63.2%) and temperature (29.1–30.2 °C) inside the boxes were automatically recorded by a humidity and temperature meter (GM1360, Shenzhen Jumaoyuan Science and Technology, Shenzhen, China). The total time the fish were in transit was approximately 48 h.

### 2.4. Recovery Time Study and Specimen Collection

Once back at PSU, Hat Yai Campus, all the fish were transferred into polypropylene cups and reared individually in the condition described above in the section headed “Fish preparation”. Non-transported fish (*n* = 15) were used as control and were sampled immediately after conditioning. Fifteen transported fish were collected for analysis immediately after transportation and labeled 0 h post-transportation (0 PT, *n* = 15). The remaining fish (*n* = 90) were reared for 12 days. During the recovery time, fish were sampled at 2, 4, 6, 8, 10, and 12 days, starved for 12 h, and anesthetized with 15 mg L^−1^ of clove oil to assess the indices. The number of fish was counted to calculate the survival rate (%) according to the equation [final fish number/initial fish number] × 100. Skin coloration was measured before biometric measurements. Fish were weighed and their lengths were measured using a vernier caliper. These biometrics were used to calculate as follows: weight loss (%) = [initial body weight (g)—final body weight (g)/initial body weight (g)] × 100, and condition factor (CF) = [live body weight (g)/standard length (cm)^3^] × 100. Visceral organs were separated and weighed for calculating viscerosomatic index (VSI, %) from the equation [wet weight of visceral organ (g)/body weight (g)] × 100. After that, visceral organs (*n* = 6), white muscle (*n* = 3), and whole carcasses (*n* = 3) were collected and used to analyse digestive enzyme activities, muscle quality, and proximate chemical composition, respectively.

### 2.5. Water Quality Parameters

The rearing water was randomly sampled every other day (*n* = 5) before being changed. Water pH (pH meter) and temperature (thermometer) were recorded. The analysis of the four parameters below followed the method of Boyd and Tucker [22]. Dissolved oxygen was measured by azide modification method. Total alkalinity and hardness were evaluated through titration with sulfuric acid and ethylenediaminetetraacetic acid, utilizing methyl orange and Eriochrome Black-T as respective indicators. Total ammonia nitrogen was assessed using the phenate method. Nitrite and nitrate were determined by colorimetric and cadmium reduction, respectively, based on the methods of APHA, AWWA, and WPCF [23]. During the experiment, the monitored physico-chemical water parameters were temperature (31.1 ± 0.3 °C, min–max = 29.0–33.0 °C), pH (6.70 ± 0.01, 6.51–7.18), dissolved oxygen (6.70 ± 0.10 mg L^−1^, 5.60–8.00 mg L^−1^), total alkalinity (26.3 ± 0.9 mg L^−1^, 16.0–32.0 mg L^−1^), hardness (66.0 ± 2.5 mg L^−1^, 40.0–96.1 mg L^−1^), total ammonia nitrogen (1.13 ± 0.05 mg L^−1^, 0.77–2.14 mg L^−1^), nitrite (0.02 ± 0.01 mg L^−1^, 0.00–0.24 mg L^−1^), and nitrate (1.31 ± 0.12 mg L^−1^, 0.00–2.14 mg L^−1^).

### 2.6. Bubble-Nest Creation

To enable the observation of bubble-nest activity, all fish of each treatment (*n* = 15) were placed with its fish inside a box that provided a black background illuminated with an off-white light-emitting diode. A smartphone (iPhone 13 Pro, Apple Inc., Zhengzhou, China) was used to take top-view photographs of the containers. The acquired images were processed using Image J software (Version 1.54g 18 October 2023, National Institutes of Health, Bethesda, MD, USA) to compute the total area of bubble-nest (cm^2^) in each container.

### 2.7. Skin Coloration

The measurement of skin color was performed in all fish of each treatment (*n* = 15) with a spectrophotometer (MiniScan EZ, Hunter Associates Laboratory, Reston, VA, USA) focused on an area in the middle of the fish. The equipment was calibrated with the white and black standard. The color parameters were automatically recorded in terms of lightness (L*), redness (a*), yellowness (b*), chroma (C*), hue (h*), and redness index (a*/b*). The value L* indicates darkness (0) to brightness (100), a* indicates the degree of redness (+*a*) to greenness (−*a*), b* indicates the degree of yellowness (+*b*) to blueness (−*b*), C* indicates color purity from vivid (+*C*) to dull (−*C*), and h* indicates color differences due to wavelength from red (0°) to blue (270°).

### 2.8. Determination of Digestive Enzyme Activities

The catabolism of proteins, lipids, and carbohydrates during fish transportation and starvation was investigated via digestive enzyme activity. Gastrointestinal tracts of the fish samples were frosted and homogenized in a cold 0.2 M Na_2_HPO_4_-NaH_2_PO_4_ buffer (pH 8) at a weight-to-volume ratio of 1:20, employing a tissue micro-homogenizer (THP-220; Omni International, Kennesaw, GA, USA). Homogenate samples were centrifuged at 15,000× *g* at 4 °C for 30 min. The supernatant was gathered and preserved in small aliquots at –20 °C.

All assays were carried out within one month of extraction. Pepsin activity was assessed following the procedure described by Worthington [24], utilizing hemoglobin as the substrate. One unit (U) of enzyme was defined based on an increase of 1.0 in absorbance at 280 nm. The activities of trypsin and chymotrypsin were determined following the method outlined by Rungruangsak-Torrissen et al. [25], employing *N*-benzoyl-*L*-Arg-*p*-nitroanilide and *N*-succinyl-Ala-Ala-Pro-Phe-*p*-nitroanilide as the respective specific substrates, and comparing absorbance at 410 nm against the linear range of a *p*-nitroanilide standard curve. Alpha-amylase activity was evaluated according to the method of Areekijseree et al. [26], using soluble starch as the substrate and measuring absorbance at 540 nm relative to the linear range of a maltose standard curve. Lipase activity was assayed by the procedure outlined by Winkler and Stuckmann [27], with *p*-nitrophenyl palmitate as substrate. The liberated product was measured spectrophotometrically at 410 nm and compared to the linear range of the *p*-nitrophenol standard curve. One unit (U) of these enzymes was defined as the amount capable of catalyzing the conversion of 1 μmol of substrate per minute. The amylase/trypsin ratios (A/T ratio) were computed by dividing the activity of amylase by the activity of trypsin obtained from same fish sample.

### 2.9. Muscle Quality Determination

#### 2.9.1. Muscle Protein Synthesis Capacity

RNA and protein concentrations were assessed using the procedure outlined by Rungruangsak-Torrissen [14] with certain adjustments. To summarize, frosted white muscle (~30 mg) was sonicated with TRIzol^®^ reagent (Invitrogen, Carlsbad, CA, USA) and mixed with chloroform to separate the upper aqueous phase containing RNA and the lower organic phase containing protein. Subsequently, both phases underwent precipitation with isopropanol and washing with ethanol. The aqueous phase was then heated to dryness in an oven and dissolved in sodium acetate. Absorbance was measured at 260 nm. The organic phase was dissolved in sodium dodecyl sulfate and its absorbance was measured at 280 nm. The extinction coefficients for calculating RNA and protein concentrations were *E*_260_ = 40 μg RNA mL^−1^ and *E*_280_ = 2.1 mg protein mL^−1^, respectively.

#### 2.9.2. Muscle Myosin and Actin Contents

The thermal properties of betta fish muscle were investigated using a differential scanning calorimeter (DSC7; Perkin Elmer, Waltham, MA, USA), following the methodology outlined by Thongprajukaew et al. [4]. Thawed white muscle samples weighing around 10 mg were carefully placed in aluminum pans, covered with aluminum lids and heated at a rate of 5 °C min^−1^ from 20 to 100 °C relative to an empty pan. The objective was to identify myosin and actin proteins from the onset (T_o_), peak (T_p_), and conclusion (T_c_) temperatures in the obtained thermograms. Additionally, the energy required for the denaturation of each protein at specific temperatures was calculated. These values were automatically converted to native protein contents based on the area under the curve.

### 2.10. Analysis of Whole-Body Composition

Before analysis, whole-body specimens underwent uniform pulverization to ensure sample homogeneity for the assessment of proximate compositions, as outlined in the AOAC [28] standards. The assessment considered moisture, crude protein, crude lipid, and ash contents. To evaluate moisture, content samples were dried for 24 h at 105 °C in a hot air oven (WOF155; Wisd Laboratory Instruments, Wertheim, Germany). Crude protein content was determined using the Kjeldahl analyzer (Kjeltec™ 8100; Foss, Höganäs, Sweden). Crude lipid content was assessed using a Soxhlet extraction unit (Soxtec™ 8000; Foss, Suzhou, China) with petroleum ether as the solvent. Ash content was determined through gravimetric analysis by incinerating samples at 600 °C for 2 h in a muffle furnace (E30-HT; Thai Furnaces Engineering, Lampang, Thailand). These analytical methods were also applied to evaluate the proximate composition of the experimental diet.

### 2.11. Statistical Analysis

The appropriate sample size per treatment was established using R version 3.6.0 to attain a test power of 0.8. Our research adopted a completely randomized design with seven distinct recovery times and experimental units of 15 fish. Arcsine transformation was applied to variables that are percentages. The homogeneity and normality of variance were examined. The achieved data were presented as means ± standard errors (SE). Statistical analyses were performed using the Statistical Package for Social Sciences version 22 (SPSS Inc., Chicago, IL, USA). Variations among means across treatment groups were evaluated through a one-way analysis of variance, using Duncan’s multiple range test as a post hoc test.

## 3. Results

### 3.1. Survival, Morphometrics, and Growth Recovery

Post-transportation time had no effect on survival and VSI (Table 1, *p* > 0.05). Standard and total lengths of reared fish had increased significantly in the 8 PT group (*p* < 0.05). The recovery in growth, as indicated by final body weight, increased with post-transportation time (*p* < 0.05) and led to a significant reduction in WL and proportionally stable CF from 8 PT until the end of observation (*p* > 0.05).

### 3.2. Bubble-Nest Creation Ability

**Figure 1:**
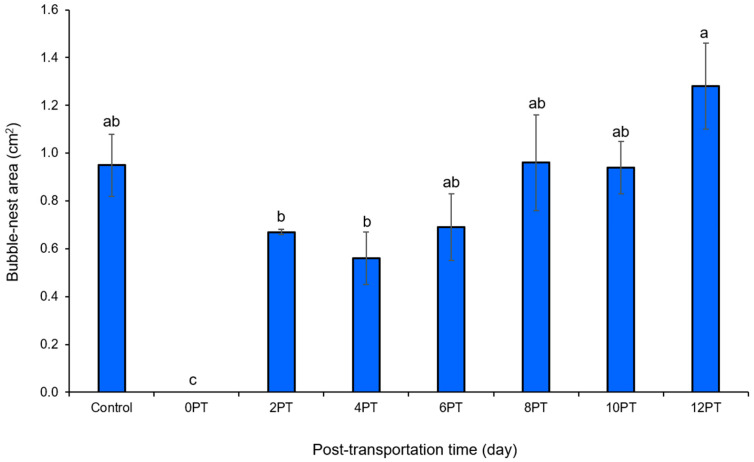
Bubble-nest area of male Siamese fighting fish after 48 h road transportation. Specimens (*n* = 15) were harvested on days 0, 2, 4, 6, 8, 10, and 12 after transportation. Fish in the control group were not transported. Different superscript letters indicate a significant difference (*p* < 0.05) between treatments. PT = post-transportation.

No bubble-nest activity was observed at 0 PT (Figure 1). Compared with the control, no differences were observed from 2 PT until 12 PT (*p* > 0.05).

### 3.3. Body Coloration

Post-transportation time had no effect on yellowness, hue, and redness index (Table 2, *p* > 0.05). The highest redness and chroma were observed in control fish (*p* < 0.05) but both values were the same as fish in the 8 PT group (*p* > 0.05). Except for 0 PT, similar lightness values were found across all treatments (*p* > 0.05).

### 3.4. Digestive Enzyme Activities

**Figure 2:**
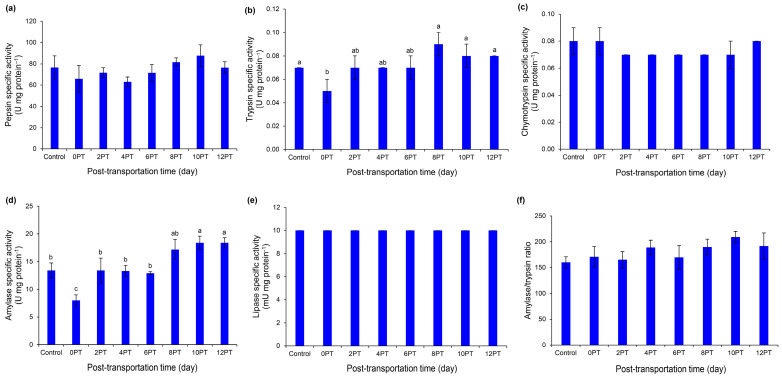
Specific activities of digestive enzymes in male Siamese fighting fish after 48 h road transportation: pepsin (**a**), trypsin (**b**), chymotrypsin (**c**), amylase (**d**), lipase (**e**), and amylase/trypsin ratio (**f**). Specimens (*n* = 5) were harvested on days 0, 2, 4, 6, 8, 10, and 12 after transportation. Fish in the control group were not transported. Different superscript letters indicate a significant difference (*p* < 0.05) between treatments. PT = post-transportation.

Post-transportation time had no effect on the specific activities of pepsin (Figure 2a, *p* > 0.05). Compared to control fish, a significant decrease in trypsin-specific activity was observed in the 0 PT treatment (Figure 2b, *p* < 0.05). Enzyme activity increased progressively until the baseline was reached at 8 PT (Figure 2b, *p* > 0.05). Although chymotrypsin-specific activities remained constant (Figure 2c, *p* > 0.05), amylase changed in a manner comparable to that of trypsin (Figure 2d, *p* < 0.05). The specific activity of lipase (Figure 2e) and amylase/trypsin ratio remained constant throughout time (Figure 2f, *p* > 0.05).

### 3.5. Muscle Quality

The highest RNA concentration was observed in the 4 PT group, followed by the 6 PT and 8 PT groups (Table 3, *p* < 0.05). No differences were observed in amounts of protein and RNA/protein ratios across all treatments (*p* > 0.05). There were no differences in amounts of myosin, actin, and their summation and ratios (*p* > 0.05).

### 3.6. Whole-Body Composition

Post-transportation time had no effect on whole-body proximate composition (Table 4, *p* > 0.05). Fish in all treatments comprised 74.4% moisture, 14.8% crude protein, 2.90% crude lipid, and 4.68% ash.

## 4. Discussion

Siamese fighting fish are frequently transported commercially overland, although little is known about the growth recovery period following transit. According to our investigations, fish were able to regain growth on the eighth day following two days of overland transit. Research conducted on yellowfin seabream (*Acanthopagrus latus*) [29], Siberian sturgeon (*Acipenser baerii*) [30], and beluga (*Huso huso*) [31] demonstrated total growth compensation through increased weight gain and final body weight after a regimen of two days of starvation followed by eight days of refeeding. Their findings are supported by the present study, which showed that compensatory growth in fish in the form of significantly increased body weight and decreased WL can result from a low basal metabolism, increased feed intake (hyperphagia), or enhanced feed utilization indices [32,33,34]. It is plausible that the primary mechanism that drove the compensatory response observed in the 8 PT group was improved feed efficiency without hyperphagia, similar to findings in Siberian sturgeon [30]. However, various factors such as species, size, health status, nutritional status before feed restriction, physiological characteristics, and the duration of feed restriction during transportation might also affect recovery time. Therefore, these factors can significantly influence how fish respond to transportation stress and subsequent recovery processes.

Survival rates and somatic indices are critical indicators of the nutritional status and health of fish [29]. The survival rate of *B. splendens* in the present study was 100% from 0 PT to 12 PT and VSI was almost unchanged. These unchanged parameters suggest that the health status of *B. splendens* remained stable without experiencing acute adverse effects during recovery after transportation. Meanwhile, there was a notable decrease in CF and an increase in SL and TL observed from 8 PT until 12 PT. Similar findings of significantly decreased CF compared to controls were reported by Gallardo-Collí et al. [35] for Nile tilapia subjected to a 3-day starvation and 9-day refeeding cycle. On the other hand, the increase in SL and TL suggests that the recovery period was sufficient to positively affect physical traits, particularly the body length.

Generally, male fighting fish construct bubble-nests to facilitate the oxygenation of developing fertilized fish eggs and larvae and protect them against predators [36,37]. Moreover, increased bubble-nest building has been linked to reduced aggressiveness or stress responses, indicated by low cortisol hormonal levels [13]. This aligns with our findings, which showed a gradual increase in bubble-nest area over time following transportation. The largest nest area was observed in the 12 PT group. The observed differences may be attributed to stress and feed deprivation during transportation [9,10]. The recovery of this behavior suggests the potential for full recovery of fighting fish in terms of growth and health status. Similarly, a previous study by Thongprajukaew et al. [4] reported that fighting fish transported in the smallest water volume (40 mL) experienced high stress that was demonstrated by reduced bubble-nest building and swimming activity.

Indeed, betta fish can experience stress from vibrations and shocks during transportation [6], which can result in adverse changes in color triggered by responses from chromatophores in the skin. Similarly, previous studies noted a loss of skin pigmentation in orange thick-lipped gourami (*Trichogaster labiosa*) [38] and fighting fish [4] during transportation. Nevertheless, temporary changes in skin pigmentation can revert to normal if environmental conditions are suitable. Our research indicated that the redness and chroma values in betta fish were restored in the 8 PT group, showing no significant difference compared to the control group, which displayed the highest redness and chroma values. Values of yellowness, hue, and redness index remained constant across all treatments while lightness values were similar across all treatments except for 0 PT.

The duration of post-transportation recovery may have influenced protein-digesting enzymes. A significant decrease was observed in trypsin-specific activity in the 0 PT relative to the control group. However, the activity of this enzyme increased, reaching baseline levels by 8 PT. Meanwhile, the specific activities of pepsin and chymotrypsin remained consistent across all treatments because trypsin and chymotrypsin are the primary enzymes responsible for growth and feed utilization of betta fish [39]. These positives point to an increase in the efficiency of protein digestion within the gastrointestinal tract and facilitate the efficient utilization of dietary protein, which is crucial considering the carnivorous nature of betta fish. Amylase-specific activity followed a similar pattern to trypsin-specific activity, suggesting an enhanced utilization of carbohydrates compared to the 0 PT group. Furthermore, the A/T ratio was maintained across all treatments, indicating the normal utilization of available proteins per unit of carbohydrate. Lipase specific activity was stable across all treatments, suggesting regular gastrointestinal functionality in lipid digestion. Small changes in digestive enzyme activities across post-transportation times in this species might be due to a physiologically wide post-prandial pattern that maintained nutrients and enzyme flexibility in response to 48 h transportation.

White muscle serves as a storage site for metabolism and protein growth. Muscle quality metrics, such as RNA, protein, and its turnover rate (RNA/protein ratio), can be a sensitive indicator of fish condition, physiological changes, or growth performance following starvation–refeeding cycles, such as starvation during transportation and subsequent refeeding [15,16,39]. Our findings revealed that growth rate post-transportation was compensated through an improved capacity to synthesize protein, with the highest RNA concentration noted at 4 PT, followed by 6 PT or 8 PT, as opposed to 0 PT. However, protein levels and RNA/protein ratios remained consistent across all treatments. This observation was corroborated by both the total amounts or proportion of the main myofibrillar proteins (actin and myosin) that remained in their original states and their unaltered amounts. These results suggest that transportation and starvation had no adverse effects on physiological movement and muscle contractility. In addition, the entire body composition was the same across the control and all other treatment groups, reinforcing that the transport did not cause a negative effect on protein metabolism and the fish recovering for 8 days solved any other transport implications. Nevertheless, the duration of required recovery periods could be affected by species, size, health condition, pre-transportation nutrition, physiological characteristics, and the duration of feed restriction during transit.

## 5. Conclusions

An optimal recovery period of eight days facilitated growth restoration and sustained fish health after shipping. During this duration, lost weight was restored, and the condition factor stabilized, indicating enhanced physiological status. Eight days after shipping, fish demonstrated equivalent bubble-nest building activity, color parameters, digestive enzyme activities, muscle quality, and whole-body composition to the control group that was not transported. These findings offer valuable insights for the management of betta fish farms, indicating an eight-day recovery period as an effective care practice for transported fish. The animal protocols for transportation, acclimatization, and future experimental procedures could be based on the recovery trajectories indicated by the data acquired in this study. Further investigations on blood cortisol, glucose, and metabolite concentrations, as well as gene expression, will advance our knowledge of the stress response in addition to the somatic response. Because there are a lot of variables involved in the transportation process, including sample preparation and packaging, transportation vehicles, routes and durations, and transportation environments, additional research is necessary to determine how these factors may impact the growth recovery time.

## Figures and Tables

**Table 1:** Survival, morphometrics, and growth recovery of male Siamese fighting fish after shipping by road. The observed parameters were recorded up to twelve days after transportation.

Parameter	Control	0 PT	2 PT	4 PT	6 PT	8 PT	10 PT	12 PT	*p*-Value
Survival (%)	100	100	100	100	100	100	100	100	−
IBW (g)	1.57 ± 0.05	1.55 ± 0.04	1.57 ± 0.06	1.58 ± 0.06	1.55 ± 0.04	1.57 ± 0.06	1.55 ± 0.04	1.57 ± 0.05	0.999
SL (cm)	3.27 ± 0.04 ^b^	3.40 ± 0.03 ^b^	3.26 ± 0.07 ^b^	3.40 ± 0.04 ^b^	3.42 ± 0.04 ^b^	3.74 ± 0.08 ^a^	3.82 ± 0.08 ^a^	3.85 ± 0.05 ^a^	<0.001
TL (cm)	5.73 ± 0.11 ^bc^	5.67 ± 0.06 ^bc^	5.52 ± 0.12 ^c^	5.81 ± 0.07 ^b^	5.87 ± 0.07 ^b^	6.33 ± 0.08 ^a^	6.18 ± 0.11 ^a^	6.19 ± 0.11 ^a^	<0.001
VSI (g)	2.88 ± 0.25	2.75 ± 0.33	2.99 ± 0.43	3.26 ± 0.32	3.09 ± 0.18	3.51 ± 0.30	2.93 ± 0.34	3.63 ± 0.48	0.561
CF (g cm^−3^)	4.52 ± 0.14 ^a^	3.33 ± 0.07 ^cd^	4.10 ± 0.25 ^ab^	3.71 ± 0.14 ^bc^	3.86 ± 0.13 ^b^	3.09 ± 0.21 ^d^	3.10 ± 0.23 ^d^	2.99 ± 0.10 ^d^	<0.001
FBW (g)	−	1.31 ± 0.04 ^d^	1.37 ± 0.05 ^d^	1.44 ± 0.04 ^cd^	1.53 ± 0.04 ^bc^	1.57 ± 0.06 ^ab^	1.65 ± 0.04 ^ab^	1.70 ± 0.04 ^a^	<0.001
WL (%)	−	15.0 ± 1.5 ^a^	12.6 ± 1.9 ^a^	4.39 ± 0.89 ^b^	4.05 ± 0.91 ^b^	−3.36 ± 0.98 ^c^	−4.68 ± 0.96 ^c^	−6.99 ± 0.98 ^c^	<0.001

PT, post-transportation time (day); IBW, initial body weight; SL, standard length; TL, total length; VSI, viscerosomatic index; CF, condition factor; FBW, final body weight; WL, weight loss. Data are expressed as means ± SE (*n* = 15 per treatment). Significant differences in each row are indicated by different superscripts letters (*p* < 0.05).

**Table 2:** Skin color coordinates of male Siamese fighting fish after shipping by road. The observed parameters were recorded for twelve days after transportation.

Parameter	Control	0 PT	2 PT	4 PT	6 PT	8 PT	10 PT	12 PT	*p*-Value
L*	24.4 ± 0.9 ^bcd^	28.5 ± 0.7 ^a^	26.4 ± 0.7 ^b^	25.6 ± 0.6 ^bc^	23.6 ± 0.5 ^cd^	24.7 ± 0.6 ^bcd^	24.4 ± 0.8 ^bcd^	23.3 ± 0.8 ^d^	<0.001
a*	12.5 ± 0.7 ^a^	7.61 ± 0.51 ^c^	9.97 ± 0.47 ^b^	9.37 ± 0.64 ^b^	10.4 ± 0.5 ^b^	11.0 ± 0.6 ^ab^	9.25 ± 0.48 ^b^	9.81 ± 0.60 ^b^	<0.001
b*	4.37 ± 0.81	2.96 ± 0.53	3.57 ± 0.37	2.54 ± 0.56	3.16 ± 0.67	4.27 ± 0.84	2.80 ± 0.41	3.42 ± 0.53	0.425
C*	13.6 ± 0.7 ^a^	8.54 ± 0.30 ^d^	10.7 ± 0.5 ^c^	9.99 ± 0.57 ^cd^	11.2 ± 0.5 ^bc^	12.4 ± 0.7 ^ab^	9.83 ± 0.41 ^cd^	10.6 ± 0.5 ^c^	<0.001
h*	0.33 ± 0.06	0.39 ± 0.08	0.34 ± 0.03	0.29 ± 0.06	0.29 ± 0.06	0.37 ± 0.08	0.30 ± 0.05	0.35 ± 0.06	0.941
a*/b*	2.81 ± 0.40	3.17 ± 0.61	3.24 ± 0.38	3.90 ± 0.76	4.09 ± 0.67	2.48 ± 0.44	3.58 ± 0.47	3.78 ± 0.77	0.527

PT, post-transportation time (day); L*, lightness; a*, redness; b*, yellowness; C*, chroma; h*, hue; a*/b*, redness index. Data are expressed as means ± SE (*n* = 15 per treatment). Significant differences in each row are indicated by different superscripts letters (*p* < 0.05).

**Table 3:** Muscle quality of male Siamese fighting fish shipped by road. The observed parameters were recorded for twelve days after shipping.

Parameter	Control	0 PT	2 PT	4 PT	6 PT	8 PT	10 PT	12 PT	*p*-Value
Protein synthesis capacity									
RNA (μg g^−1^)	3500 ± 157 ^bc^	3116 ± 291 ^c^	3351 ± 148 ^bc^	4255 ± 300 ^a^	3658 ± 22 ^abc^	3897 ± 237 ^ab^	3318 ± 26 ^bc^	3464 ± 124 ^bc^	0.017
Protein (mg g^−1^)	197 ± 23	190 ± 6	200 ± 12	223 ± 22	235 ± 12	166 ± 7	214 ± 28	193 ± 8	0.202
RNA/protein ratio (μg mg^−1^)	18.5 ± 3.1	16.4 ± 1.7	16.9 ± 1.7	19.8 ± 3.6	15.7 ± 0.8	23.5 ± 0.6	16.1 ± 2.1	18.1 ± 1.4	0.237
Muscle protein									
ΔH Myosin (J g^−1^)	1.34 ± 0.06	1.53 ± 0.28	1.02 ± 0.39	1.33 ± 0.05	1.37 ± 0.17	1.44 ± 0.22	1.43 ± 0.14	1.41 ± 0.13	0.817
ΔH Actin (J g^−1^)	0.37 ± 0.02	0.27 ± 0.04	0.26 ± 0.06	0.37 ± 0.01	0.34 ± 0.02	0.35 ± 0.08	0.29 ± 0.05	0.35 ± 0.01	0.401
ƩΔH (J g^−1^)	1.71 ± 0.04	1.80 ± 0.32	1.29 ± 0.42	1.70 ± 0.04	1.71 ± 0.19	1.79 ± 0.30	1.72 ± 0.15	1.76 ± 0.14	0.833
ΔH Actin/myosin	0.28 ± 0.03	0.18 ± 0.03	0.29 ± 0.08	0.28 ± 0.01	0.25 ± 0.03	0.24 ± 0.03	0.20 ± 0.04	0.25 ± 0.02	0.400

PT, post-transportation time (day); ΔH, enthalpy. Data are expressed as means ± SE (*n* = 3 per treatment). Significant differences in each row are indicated by different superscript letters (*p* < 0.05).

**Table 4:** Whole-body compositions (% of fresh weight) of male Siamese fighting fish after shipping by road. The observed parameters were recorded for twelve days after transportation.

Parameter	Control	0 PT	2 PT	4 PT	6 PT	8 PT	10 PT	12 PT	*p*-Value
Moisture	73.9 ± 1.0	74.5 ± 0.3	73.7 ± 0.6	74.9 ± 0.8	75.5 ± 1.1	73.7 ± 0.2	74.0 ± 1.0	74.7 ± 0.2	0.677
Crude protein	14.7 ± 0.5	15.3 ± 0.4	14.9 ± 0.2	14.7 ± 0.1	14.3 ± 0.3	15.0 ± 0.1	14.9 ± 0.4	14.6 ± 0.2	0.429
Crude lipid	3.18 ± 0.31	2.49 ± 0.37	3.16 ± 0.71	2.86 ± 0.34	2.55 ± 0.77	3.42 ± 0.19	3.08 ± 0.57	2.46 ± 0.20	0.766
Crude ash	5.34 ± 0.50	4.36 ± 0.13	4.74 ± 0.15	4.23 ± 0.11	4.81 ± 0.09	4.46 ± 0.23	4.48 ± 0.28	5.03 ± 0.48	0.185

PT, post-transportation time (day). Data are expressed as means ± SE (*n* = 3 per treatment).

## Data Availability

The data presented in this study are available on request from the corresponding author.
